# Metacognitive therapy vs. eye movement desensitization and reprocessing for posttraumatic stress disorder: study protocol for a randomized superiority trial

**DOI:** 10.1186/s13063-017-2404-7

**Published:** 2018-01-08

**Authors:** Hans M. Nordahl, Joar Øveraas Halvorsen, Odin Hjemdal, Mimoza Rrusta Ternava, Adrian Wells

**Affiliations:** 1 St. Olavs Hospital HF, Nidaros DPS, P.O. Box 3250, 7006 Trondheim, Norway; 2 Institute of Mental Health, Faculty of Medicine and Health Sciences, NTNU, PO box 8905, 7491 Trondheim, Norway; 3 Department of Psychology, Dragvoll NTNU, 7491 Trondheim, Norway; 4 School of Psychological Sciences, University of Manchester, Manchester, UK; 5 Greater Manchester Mental Health NHS Foundation Trust, Manchester, UK

**Keywords:** Posttraumatic stress disorder, Randomized controlled trial, EMDR, Metacognitive therapy

## Abstract

**Background:**

The psychological treatment of choice for patients with severe posttraumatic stress disorder (PTSD) is cognitive behavioural exposure therapy or Eye Movement Desensitisation Reprocessing (EMDR). Whilst these are the most effective treatments, approximately 30–45% of the patients show no significant improvements and follow-up data are sparse. Furthermore, a proportion of patients with severe trauma does not benefit or avoid exposure therapy due to the potential to overwhelm them. Therefore, it is necessary to search for effective methods that do not require exposure. Metacognitive therapy (MCT), a recent treatment approach to PTSD that does not require exposure, has potential strong treatment effects but so far a comparison with EMDR has not been made.

**Methods/design:**

This study is a two-arm, parallel, randomized, superiority trial comparing the effectiveness of MCT with EMDR. One hundred patients with a primary diagnosis of chronic PTSD will be included and will receive 12 sessions of one of the treatments. The primary outcome is severity of PTSD symptoms assessed with the Posttraumatic Diagnostic Scale (PDS) measured post-treatment (3 months). Secondary outcomes include symptom severity (PDS) and measures of anxiety, depression, metacognitive beliefs at 3-month and 12-month follow up.

**Discussion:**

This randomized study is the first to compare MCT with EMDR with 12-month follow-up. The study will indicate the comparative effectiveness of MCT against EMDR and the stability of effects when delivered in an outpatient clinical setting.

**Trial registration:**

ClinicalTrials.gov, NCT01955590. Registered on 24 September 2013.

**Electronic supplementary material:**

The online version of this article (doi:10.1186/s13063-017-2404-7) contains supplementary material, which is available to authorized users.

## Background

Posttraumatic stress disorder (PTSD) is a frequently occurring and often debilitating anxiety disorder resulting from exposure to trauma [1, 2]. Prolonged exposure therapy [3], trauma-focused cognitive behavioural therapy (CBT) [4] and eye movement desensitisation reprocessing (EMDR) [3] are recommended treatments for PTSD. Each of these approaches utilises exposure to trauma memories as one of the main components in the intervention. The efficacy of these treatments is supported by a large number of studies that show equivalent levels of outcome with no particular treatment evidencing superiority [4, 5]. However, it has been noted that EMDR has to some degree been compared to other active treatments for PTSD in rigorous controlled trials.

Although the majority of patients improve, a substantial proportion of patients drop out of treatment [4, 6, 7], present with residual symptoms following treatment or fail to improve [7–9]. Approximately 37–51% of patients completing therapy improve significantly [8], which leaves substantial room for improving outcomes. Furthermore, a substantial portion of the clinical trials on PTSD have major methodological limitations [10].

EMDR is based on the assumption that posttraumatic symptoms are caused by traumatic experience(s) being stored in an unprocessed way disconnected from existing memory networks [11]. In EMDR the patient is asked to focus on trauma-related imagery, negative cognitions and body sensations while simultaneously focusing their attention on a bilateral physical stimulation. The procedure in EMDR is postulated to facilitate the processing of the traumatic memory into existing memory networks [11]. A number of theories explaining the potential mechanisms underlying the effects EMDR have been proposed (e.g. [12]). The importance of bilateral eye movements is often highlighted, but there is still substantial controversy about whether bilateral eye movements are of importance [13] or not [14–16]. It is also important to note that dismantling randomized controlled trials comparing EMDR with and without bilateral eye movements does not in itself provide sufficient evidence on potential mediators and mechanisms for treatment outcome [17]. To the best of your knowledge, to date no RCTs have been conducted in EMDR that incorporate the investigation of potential mediators of treatment outcome as outlined by Kazdin [17, 18]. As such there is still a pressing need for trials investigating potential mediators of treatment outcome of trauma-focused treatments generally and EMDR specifically [19]. EMDR is usually considered an evidence-based treatment for PTSD [5, 8, 20].

Metacognitive therapy (MCT) [21, 22] is one of the more recent approaches in the treatment of PTSD. The focus of MCT is on removing specific barriers to spontaneous recovery that is purported to normally occur following trauma. Metacognitive beliefs are beliefs and theories people have about their thinking and how to regulate their thoughts. It could be beliefs that thoughts have a special meaning (sinful thoughts) or that thoughts may be harmful or uncontrollable. The metacognitive beliefs are hypothesised to underlie an unhelpful response style consisting of worry, rumination and threat monitoring, and other coping behaviours such as attempting to fill gaps in memory. These response styles prevent cognition and arousal returning back to basal levels of processing a threat-free environment. In effect PTSD symptoms are maintained because these factors prolong threat-related processing and interfere with the downregulation of subcortical arousal. In contrast to EMDR, MCT does not involve prescribed exposure exercises or restructuring of negative trauma-related memories.

MCT has been shown in early trials to be an effective, time-limited (8–10 sessions), well-tolerated and apparently highly effective treatment option for both recent onset and chronic PTSD [23–26]. The core treatment has been investigated using an A-B direct replication series [23], an open trial [24] and a randomized controlled trial with delayed treatment as a control condition [25]. These preliminary results indicate that MCT is a well-tolerated treatment and that most patients experience significant improvements that are maintained at follow up. Furthermore, a clinical trial to compare MCT with prolonged exposure (PE) has shown that MCT performs better [26]. This trial demonstrated a statistically significant reduction in reported PTSD symptomatology, depression and anxiety symptoms post-treatment in the MCT group compared to the PE condition. In conclusion, MCT appears to be a highly promising treatment for PTSD, generating large clinically significant treatment effects for clients exposed to a range of traumatic experiences, which include accident survivors and assault and rape victims. However, to date no study has directly compared MCT with EMDR. The aim of the present study was to undertake such a comparison to determine the relative effectiveness for each of the treatments immediately following treatment and at 12-month follow-up.

The following aims and hypotheses were set up in the trial: (1) to evaluate the effectiveness of MCT compared with EMDR in PTSD using a comparative randomized controlled study; (2) to examine potential moderators and mediators of treatment outcome; (3) to examine and compare the relapse rate in MCT and EMDR.

The main hypotheses to be tested include the following:

1. Both treatments will be effective and demonstrate significant improvements in symptom severity indicated by decreased scores for trauma symptoms, anxiety level and depressive symptoms
2. Previous findings on effect sizes [27] indicate that MCT will demonstrate higher recovery rates than EMDR based on the symptom criteria of the Posttraumatic Diagnostic Scale (PDS).

## Methods

### Study design

**Fig. 1:**
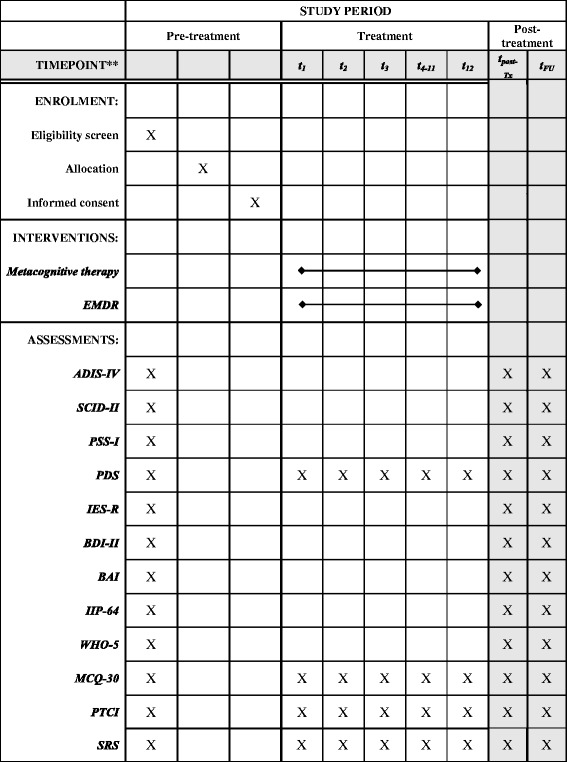
Schedule of enrolment, interventions, and assessments. *Abbreviations*: *ADIS-IV* Anxiety and Depression Interview Scale-IV; *BDI* Beck Depression Inventory; *BAI* Beck Anxiety Inventory; *IES-R* Impact of Event Scale- revised; *IIP-64* Inventory of Interpersonal Problems-64; *PDS* Posttraumatic Diagnostic Scale; *MCQ-30* Metacognitive Questionnaire-30; *PSS-I* Posttraumatic Stress Disorder Scale-Interview; *PTCI* Post Traumatic Cognitions Inventory; *SRS* Session Rating Scale

The study is a two-arm randomized controlled superiority trial with a pre-post and 12-month follow up comparing the effectiveness of MCT and EMDR in the treatment of PTSD. The primary hypothesis is that MCT will be superior to EMDR in reducing PTSD symptoms measured with the PDS post-treatment. The secondary hypothesis is that superiority of MCT will be maintained at 12-month follow up. Stratified randomization where equal numbers of participants are allocated to each condition according to chronicity of PTSD, presence and severity of borderline personality traits (four criteria fulfilled) and gender will be used. The independent variable is a manualised MCT condition (12 sessions) or manualised EMDR (12 sessions). The other secondary outcome measures are the Impact of Events Scale-revised (IES-R), posttraumatic symptoms assessed using the Posttraumatic Stress Scale-Interview (PSS-I), depression assessed using the Beck Depressions Inventory (BDI)-II, anxiety levels assessed using the Beck Anxiety Inventory (BAI)-II, and metacognitions assessed using the Metacognitive Questionnaire (MCQ)-30). The present protocol has been prepared in accordance with relevant items from the SPIRIT Checklist (see Additional file 1) and the SPIRIT Figure (Fig. 1).

### Participants

Eligible patients who meet *Diagnostic and Statistical Manual of Mental Disorders-Fourth Edition* (DSM-IV) criteria for PTSD and are aged between 16 and 70 years of age and consent to participation will be included. Diagnosis will be determined with the Anxiety Disorders Interview Schedule (ADIS-IV). Patients must not have previously received EMDR or MCT. Patients with psychotic symptoms, severe depression, alcohol or drug abuse, acute suicidality, borderline personality disorder or symptoms of PTSD for less than 6 months will be excluded.

### Measures

Both clinical assessments and self-report measures will be used in this study. Figure 1 shows the time distribution of each rating or measure (see Fig. 1).

#### Posttraumatic Diagnostic Scale *(PDS)*

The PDS [28] is a self-report diagnostic and symptom severity measure of PTSD based on the DSM-IV criteria. It consists of a total of 49 items. Of these, 17 items are related to diagnostic criteria as defined by the DSM-IV and these are rated on a 4-point Likert scale. The symptom severity score (PDS-SSS) was used to indicate severity of symptoms. Test-retest reliability over 3 weeks for the diagnosis of PTSD was acceptable (k = 0.74) and the validity is confirmed by high overlap between PDS and the structured clinical interview for PTSD (82%) [28].

#### Anxiety Disorders Interview Schedule *(ADIS)*

The ADS [29] will be used as the diagnostic measure. This is a structured clinical interview based on the DSM-IV criteria.

#### PTSD Symptom Scale – Interview *(PSS-I)*

The PSS-I [30] was designed as a flexible semi-structured interview about DSM-IV PTSD symptoms to make a diagnosis of PTSD and obtain an estimate of the severity of the symptoms. PTSD severity is determined by the sum score of the 17 PSS-I item ratings. Scores range from 0 to 51. PTSD diagnosis is determined by counting the number of symptoms endorsed (a rating of 1 or greater) per symptom cluster: 1, re-experiencing; 3, avoidance; and 2, arousal symptoms are needed to meet diagnostic criteria. The PSS-I has been compared to the Clinician-Administered PTSD scale (CAPS) and they correlated strongly with each other and with the Structured Clinical Interview for DSM Disorders (SCID) [31]. These results indicate that PSS-I can be used in the same way as CAPS in the assessment of PTSD.

#### Beck Anxiety Inventory (*BAI)*

The BAI [32] is an inventory designed to assess the broad spectrum of anxiety symptoms and consists of 21 items. The respondents score the degree to which they have been impaired by the symptoms during the last week. Total scores range from 0 to 63, and a higher score indicates greater levels of anxiety. The BAI has high internal consistency and test-retest reliability.

#### Beck Depression Inventory *(BDI- II)*

The BDI-II [33] will be used as a measure of depressive symptom severity. The BDI-II ranges from 0 to 63 and has an alpha of 0.94, and test-retest reliability of 0.93.

#### Impact of Event Scale – revised *(IES-R)*

The IES-R [34] is one of the most commonly used measures of PTSD symptoms [35]. The respondents rate the frequency of the subjective experience of distress in relation to their traumatic event within the last week. It consists of two subscales for intrusion and avoidance. The psychometric properties are well-validated [35, 36].

#### Metacognitions Questionnaire-30 *(MCQ-30)*

The MCQ-30 [37] is a 30-item self-report questionnaire used to measure metacognitive beliefs. It consists of five subscales; negative beliefs about uncontrollability of thoughts and danger; positive beliefs about worry; beliefs about need to control thoughts; cognitive confidence and cognitive self-consciousness. Higher scores indicate higher levels of maladaptive metacognition. The validity and reliability of MCQ-30 is well-established in adults [37].

#### Inventory of Interpersonal Problems *(IIP-64-C)*

This self-report measure [38] consists of 64 items: 39 items begin with: “It is hard for me to …” and 25 items introduce “Things that you do too much”. Each item is rated on a 5-point Likert scale, ranging from 0 (not at all) to 4 (extremely). The inventory is tapping eight specific interpersonal problem areas. Many studies have estimated Cronbach’s alpha for the IIP-64 scales in the range 0.72–0.85 [38–40], and test-retest correlation in the range of 0.56–0.83 [41].

#### Session Rating Scale *(SRS)*

The SRS [42] is a four-item visual analogue instrument designed to measure the quality of the working alliance on a session-to-session basis. Item analysis of the SRS provided a Cronbach’s alpha reliability coefficient of 0.88, and a test-retest reliability of 0.64.

#### The Posttraumatic Cognitions Inventory *(PTCI)*

The PTCI [43] is a widely used instrument to assess cognitions deemed to be important in the development and maintenance of posttraumatic symptoms. It consists of 36 items scored on a 7-point Likert scale. The instrument has excellent psychometric properties [43].

#### The WHO-5 Well-Being Index *(WHO-5)*

As a measure of overall psychological well-being we use the WHO-5 [44]. The instrument consists of five items measured on a 6-point Likert scale. The WHO-5 scale is considered a generic scale for the measurement of general well-being [45] and is deemed to have acceptable psychometric qualities [44] with a Cronbach coefficient alpha of 0.84 [46].

#### Sample size estimation

We assume similar post-treatment scores on the PDS as reported by Wells and colleagues [26] in their comparison of MCT with prolonged exposure, where M = 10.4 for MCT and M = 18.3 for the PE condition, with a pooled standard deviation of 10.6. It is estimated that for a two-tailed superiority trial, 58 patients are required in total (29 per arm) to have an 80% chance of detecting a significant difference in the primary outcome measure at the 5% level. We will aim to recruit more patients than this in order to compensate for dropouts.

### Procedure

The trial will be conducted at the specialist clinic for PTSD and traumatic stress at Nidaros DPS, St. Olavs hospital. Patients referred to the clinic will be screened and assessed for eligibility. Patients meeting the inclusion criteria will be invited to participate in the trial, and consent will be obtained by signing the form. Patients referred to the clinic who either (a) decline to participate in the trial or (b) do not meet the inclusion criteria but suffer from trauma-related symptomatology, will be given appropriate treatment as usual (TAU) at the clinic. Patients referred to the clinic who are not in the clinic target group, i.e. patients not suffering from trauma-related mental health problems, will be referred to other appropriate services.

The initial assessment will take approximately 90 minutes and will consist of carrying out a number of measures. Initially the Mini International Neuropsychiatric Interview (MINI) [47] will screen patients for PTSD symptoms and co-morbid Axis I disorders. If the inclusion criteria are met, then the above-described instruments will be administered, including those to assess primary PTSD symptoms, anxiety symptoms and comorbid mood disorders. Patients meeting the inclusion criteria and consenting to participate in the trial will be randomly allocated to the MCT condition or EMDR condition.

### Randomization

Participants meeting the inclusion criteria are randomized to either EMDR or MCT. Randomization is stratified on gender and borderline personality disorder characteristics, as both gender and BPD characteristics have been found/are assumed to impact treatment outcome. Before initiating the trial the principal investigator (HMN) generated a random-number table using SPSS. Due to the use of a printed random-number table the allocation is not absolutely/perfectly concealed. Authors HMN and JØH assign allegeable participants to the conditions based on the pre-defined random-number table.

### Blinding

We aimed to make the assessors blind to the treatment the participants had received by assembling a team of assessors not involved in the treatment of the participants. However, since both therapists and assessors worked at the same outpatient clinic it is difficult to ascertain whether the assessors are indeed blind to the treatment the participants have received. However, due to logistical issues, two psychologists assigned to the assessor-team treated a couple of the participants in the trial. In these cases the assessors and the therapists were independent of each other, such as that a psychologist could not be the assessor and the therapist for the same patients.

### Plans to promote participant retention and complete follow-up

We will strive to collect data on all participants irrespective of whether they complete treatment or not. Data will primarily be collected through an electronic system/platform delivered by CheckWare (http://checkware.com/en). CheckWare is an internet-based system/platform for collecting, processing and saving sensitive information such as patient self-reports in a secure way. We believe this solution will ease the burden of self-report for the participants and facilitate the data collection. For participants who are unwilling or unable to make use of CheckWare we will provide the usual pen and pencil self-report forms. Participants will receive a notification delivered by SMS when it is time to complete 6-month and 12-month follow-up self-report assessments. Participants who do not complete these assessments will be contacted by telephone to assist them with any obstacles with completing the assessments. We will send an abbreviated pen and pencil version of the self-report package by ordinary mail to participants that do not respond to either the SMS notification or to the telephone call. Participants who are still unresponsive will be offered the possibility to come to the clinic to meet their clinician for more personalized follow-up, which includes completing an abbreviated pen and pencil version of the self-report package.

### Treatments

MCT will be based on the published treatment manual developed by Wells [21, 22] consisting of up to 12 sessions of individual treatment. Clients allocated to the EMDR condition will complete 12 sessions based on the structured protocol developed by Shapiro [11]. Sessions for both treatments will be held on a weekly basis and will initially last 45–60 minutes. Participants will be asked to complete further assessments to monitor their progress at the mid-point of therapy, end of treatment and 12 month follow-up. We chose EMDR as a comparator for two primary reasons: First, EMDR is usually regarded as an evidence-based treatment for PTSD to which MCT has not previously been compared. Second, at the clinic where the trial is conducted, EMDR is already an established and widely used treatment. The session content for both conditions is prescribed in detail and manualised.

MCT consists of session 1: case conceptualisation and socialisation; session 2–3: detached mindfulness and worry/rumination postponement and commencement of challenging negative beliefs about symptoms and positive beliefs about worry and rumination; session 4–5: generalising techniques and banning gap-filling and other coping strategies with work on residual beliefs; session 6–7: attentional modification; session 8–9: utilising techniques and work on residual maladaptive coping strategies and beliefs; and session 10–12: developing and consolidating a therapy blueprint and new plan for guiding cognition and action in response to intrusions and symptoms.

The sessions in EMDR are session 1: history and forming an alliance; session 2–3: preparation and target for exposure, including psychoeducation and creating a safe place; session 4–10: desensitisation and reprocessing, which in essence consists of imaginal exposure to the trauma memory and cognitive restructuring of trauma-related cognitions and floatback; and session 11–12: plan relapse prevention, arrange follow up.

### Treatment adherence and fidelity

The therapists delivering the therapies are clinical psychologists who are trained in trauma-focused therapy and have at least 2 years experience. The therapists delivering MCT will be trained in MCT by the originator of the treatment (AW). They will also receive weekly supervision from HMN. The therapists delivering EMDR are all certified level-2 EMDR practitioners who have completed at least EMDR level-2 training and will be supervised by Bjørn Aasen, who is an EMDR Europe Approved Senior Trainer. Adherence to the treatments and treatment fidelity will be monitored and assessed using session by session treatment checklists completed by the therapists and monitored in supervision. Rating of checklists will assess the presence of specific therapy components and the absence of prohibited components in both treatments. All treatment sessions will be videotaped and a random sample of 10% of the tapes stratified by session and groups will be rated by experts in the treatments to determine adherence and competency.

### Concomitant care

Participants enrolled in the trial are allowed to continue psychopharmacological treatment as long as they maintain a stable dose throughout the trial. No other concomitant care will be allowed during the trial.

### Data analysis plan

All data will be analysed based on an intention-to-treat (ITT) approach with all randomized patients entering the analysis. Missing data will be estimated by SPSS multiple imputation on the primary measure to estimate the scores of those with missing data. The number of imputations will depend on the amount of missing data. The primary outcome is a continuous longitudinal measure (PDS), therefore linear mixed-model analysis (LMM) will be run. Random effects will be applied to the patient-specific intercepts and covariates will be included in all analyses based on imbalance between trial arms. The effects of the treatments will be examined using the interaction effect of group × time from the mixed-effects analysis with PDS post-treatment as the primary outcome.

In addition to the primary analyses we will conduct exploratory moderation and mediation analyses. Moderation analyses will be conducted with both individual and combined moderators [48, 49]. Mediation analyses [50, 51] will be conducted with PDS as the outcome and PTCI, MCQ-30 and SRS as potential mediators.

## Discussion

The main aim of this study is to test the efficacy of MCT, a non-exposure protocol-based intervention, against EMDR, which is an exposure protocol-based intervention. MCT is a new and effective treatment for a range of emotional disorders, and was more effective than prolonged exposure in a recent study [26]. It may therefore prove to be more efficient than other exposure-based interventions, such as EMDR. Furthermore, MCT does not use exposure or reliving of trauma memories and this could be advantageous in reducing the aversiveness to the treatment by both the patient and the therapist.

The challenges of the study are that we have open follow up, meaning that patients who do not feel they have recovered or still are not asymptomatic may, and can, seek additional treatment after the study treatment. This could bias our 12 month follow-up assessment, thus we need to evaluate these aspects in the 12-month follow-up assessment.

Also, it may be a challenge to handle the attrition rates, which is shown to be a problem in many trials. Attrition can be responsible for biased estimates of the treatment effects. It has been reported to be between 20 and 25% in PTSD trials [10]. There is an important distinction between a participant who terminates a randomly assigned study intervention, but continues to complete the assessment measures, and a participant who ceases all study participation, including assessments. It is only the latter who has truly dropped out of the study, thereby contributing to attrition. We have developed a plan to stimulate the participant to carry on in the study to complete as many assessment forms as possible by maintaining contact during the follow-up phase.

### Trial status

Recruitment is ongoing and the treatment phase is expected to be completed by the end of 2017.

## Additional file

Additional file 1: The SPIRIT Checklist. (DOC 121 kb)

## Abbreviations

ADIS-IV: Anxiety and Depression Interview Scale-IV
ANOVA: Analysis of variance
BDI: Beck Depression Inventory
BAI: Beck Anxiety Inventory
CAPS: Clinician-Administered PTSD scale
DSM-IV: *Diagnostic and Statistical Manual of Mental Disorders-Fourth Edition*
EMDR: Eye movement desensitisation reprocessing
ITT: Intention to treat
IES-R: Impact of Event Scale-revised
LMM: Linear mixed model
MCT: Metacognitive therapy
MCQ-30: Metacognitive Questionnaire-30
Mini: Mini International Neuropsychiatric Interview
PSS-I: Posttraumatic Stress Scale-Interview
PTSD: Posttraumatic stress disorder
SCID: Structured Clinical Interview for DSM Disorders
TAU: Treatment as usual
