# Correction to: Metacognitive therapy vs. eye movement desensitization and reprocessing for posttraumatic stress disorder: study protocol for a randomized superiority trial

**DOI:** 10.1186/s13063-018-2536-4

**Published:** 2018-03-06

**Authors:** Hans M. Nordahl, Joar Øveraas Halvorsen, Odin Hjemdal, Mimoza Rrusta Ternava, Adrian Wells

**Affiliations:** 1 St. Olavs Hospital HF, Nidaros DPS, P.O. Box 3250, 7006 Trondheim, Norway; 2 Institute of Mental Health, Faculty of Medicine and Health Sciences, NTNU, PO box 8905, 7491 Trondheim, Norway; 3 Department of Psychology, Dragvoll NTNU, 7491 Trondheim, Norway; 4 School of Psychological Sciences, University of Manchester, Manchester, UK; 5 Greater Manchester Mental Health NHS Foundation Trust, Manchester, UK

## Correction to: Trials (2018) 19: 16. https://doi.org/10.1186/s13063-017-2404-7

In the original publication of this article [1] the SPIRIT figure for the study protocol was not included. In this Correction Article the SPIRIT figure (Fig. 1) is published. The original publication has been updated with the SPIRIT figure.

**Fig. 1:**
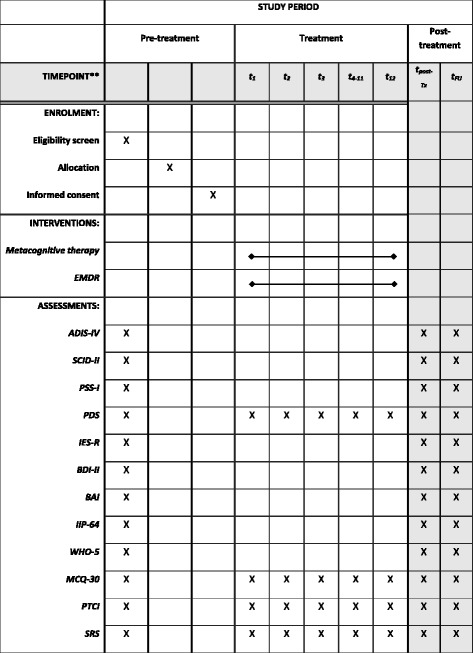
Schedule of enrolment, interventions, and assessments. Abbreviations. ADIS-IV: Anxiety and Depression Interview Scale-IV; BDI: Beck Depression Inventory; BAI: Beck Anxiety Inventory; IES-R: Impact of Event Scale- revised; IIP-64: Inventory of Interpersonal Problems-64; PDS: Posttraumatic Diagnostic Scale; MCQ-30: Metacognitive Questionnaire-30; PSS-I: Posttraumatic Stress Disorder Scale-Interview; PTCI: Post Traumatic Cognitions Inventory; SRS: Session Rating Scale
